# Data-driven identification of key predictors of uncontrolled hypertension: A cross-sectional study

**DOI:** 10.1371/journal.pone.0331565

**Published:** 2025-09-04

**Authors:** Oliver Mendoza-Cano, Xóchitl Trujillo, Miguel Huerta, Mónica Ríos-Silva, Agustin Lugo-Radillo, Jaime Alberto Bricio-Barrios, Verónica Benites-Godínez, Herguin Benjamin Cuevas-Arellano, Juan Manuel Uribe-Ramos, Ramón Solano-Barajas, Jesús Venegas-Ramírez, Eder Fernando Ríos-Bracamontes, Luis A García-Solórzano, Arlette A Camacho-delaCruz, Efrén Murillo-Zamora

**Affiliations:** 1 Facultad de Ingeniería Civil, Universidad de Colima, Coquimatlán, Colima, Mexico; 2 Centro Universitario de Investigaciones Biomédicas, Universidad de Colima, Colima, Mexico; 3 Facultad de Medicina, Universidad de Colima, Colima, México; 4 SECIHTI—Facultad de Medicina y Cirugía, Universidad Autónoma Benito Juárez de Oaxaca, Oaxaca, Mexico; 5 Coordinación de Educación en Salud, Jefatura de Servicios de Prestaciones Médicas, Instituto Mexicano del Seguro Social, Tepic, Nayarit, Mexico; 6 Unidad Académica de Medicina, Universidad Autónoma de Nayarit, Tepic, Nayarit, Mexico; 7 Facultad de Ciencias, Universidad de Colima, Colima, Mexico; 8 Coordinación Auxiliar de Investigación en Salud, Jefatura de Servicios de Prestaciones Médicas, Instituto Mexicano del Seguro Social, Colima, Mexico; 9 Departamento de Medicina Interna, Hospital General de Zona No. 1, Instituto Mexicano del Seguro Social, Villa de Álvarez, Colima, Mexico; 10 Tecnológico Nacional de México, Campus Colima, Villa de Álvarez, Colima, México; 11 Unidad de Investigación en Epidemiología Clínica, Instituto Mexicano del Seguro Social, Villa de Álvarez, Colima, Mexico; Tribhuvan University Institute of Medicine, NEPAL

## Abstract

Uncontrolled hypertension (HTN) increases the risk of adverse health events. This study aimed to identify key predictors of uncontrolled HTN in 1,308 Mexican adults with a prior diagnosis of HTN who were undergoing pharmacological treatment. We utilized data from the 2022 National Health and Nutrition Survey and applied data-driven algorithms within an artificial intelligence framework to enhance predictive accuracy and interpretability. Specifically, we integrated Random Forest, XGBoost, LASSO regression, and SHAP analysis. Uncontrolled HTN was defined as systolic blood pressure ≥ 130 mmHg or diastolic blood pressure ≥ 80 mmHg based on two readings. We applied LASSO regression to exclude unrelated factors and trained Random Forest and XGBoost algorithms to identify the most important predictors. The key contributors to model accuracy in Random Forest were years since HTN diagnosis (11.9), age (9.4), and source of medical care (4.6), while SHAP analysis in XGBoost further highlighted age (0.115) and source of medical care (0.065) as significant factors. When compared to a traditional logistic regression model, the data-driven approach demonstrated superior predictive performance, with Random Forest achieving an AUC of 0.75 (95% CI 0.72–0.77) versus logistic regression (AUC = 0.61, 95% CI 0.59–0.64). XGBoost exhibited lower predictive capacity (AUC = 0.54, 95% CI 0.49–0.60). These findings underscore the importance of age, duration since diagnosis, and source of medical care in predicting uncontrolled HTN. If replicated, this evidence can inform public health strategies to better target at-risk populations and optimize HTN management through data-driven interventions.

## Introduction

Arterial hypertension (HTN) is a major public health challenge worldwide. Despite advances in medical treatments and interventions, a subset of patients with the disease remains uncontrolled, posing increased risks of cardiovascular events, kidney disease, and mortality [1]. This results from the complexity of HTN management, which is compounded by numerous factors influencing blood pressure control, including lifestyle, comorbidities, socioeconomic status, and others [2,3].

In Mexico, HTN affects nearly half (49.4%) of adults aged 20 years and older, with prevalence increasing significantly in older age groups. Sex-based disparities exist, with higher rates among males (55.3%) compared to females (44.0%), highlighting the need for targeted interventions [4].

Artificial intelligence (AI) assisted data-driven models have offered the capability to identify complex patterns and generate predictive insights that surpass traditional statistical methods [5]. One of these models are the Random Forest algorithms, which are ensemble learning methods that construct decision trees during training and output the mode of the classes (classification) or mean prediction (regression) of the individual trees [6]. These algorithms are useful in health research due to their robustness against overfitting, their ability to handle large datasets with high dimensionality [7]. By aggregating the results of multiple trees, Random Forest provide insights into variable importance, making them a powerful tool for identifying key predictors of health outcomes such as uncontrolled HTN.

In Mexico, the National Health and Nutrition Survey (ENSANUT) 2022 collected useful data to understand health trends, identify public health challenges, and inform policy decisions aimed at improving the health and well-being of communities [8]. The survey is nationally representative, which ensures that the findings accurately reflect the health status of the entire population.

This study aimed to identify key predictors of uncontrolled HTN in adults using a data-driven approach that integrates traditional statistical methods with machine learning models. By utilizing these algorithms in health data, we may foster a more tailored and effective approach to HTN management.

## Methods

### Study design and population

We conducted a cross-sectional analysis using data from ENSANUT 2022. For this study, we selected 1,308 adults aged 20 years or older who reported a previous diagnosis of HTN, were currently taking medication to control high blood pressure, and had complete data for the variables of interest. HTN history was assessed with the question: “Has any doctor ever told you that you have high blood pressure?” with three response options: No, Yes, and Yes (during pregnancy). We excluded pregnancy-related cases from our study. The current use of medication to control blood pressure was determined by the question, “Are you currently taking any medication (pills) to control your high blood pressure?” with response options of No or Yes.

### Outcome

The primary outcome was whether adults with a previous diagnosis of HTN had uncontrolled blood pressure (No/Yes). In the survey, blood pressure was measured three times using an automated and calibrated digital device. The first reading was taken after at least 5 minutes of rest, with the second and third readings taken 2–3 minutes apart. The average of the second and third readings determined if blood pressure was uncontrolled, defined as systolic pressure ≥ 130 mmHg or diastolic pressure ≥ 80 mmHg.

### Data collection

Factors evaluated in our analysis included sex (female/male), age (years), socioeconomic status (low, middle, high), years since HTN diagnosis (≤ 5, 6–10, > 10), personal history of diabetes mellitus (assessed with the question: “Has a doctor ever told you that you have diabetes or high blood sugar?”, No/Yes), usual source of medical care for HTN (social security institutions, public health sector, private care, or other), adherence to a nutritional plan (No/Yes), and engagement in physical activity (No/Yes). These variables were selected in accordance with their association with the analyzed outcome [9–16].

The socioeconomic index, in the ENSANUT 2022, is a composite measure incorporating housing characteristics, basic services, as well as household assets [17].

### Statistical analysis

Summary statistics were computed to describe the study sample. No data imputation was performed; only adults with complete information for the variables of interest were included in the analysis. To identify key predictors of uncontrolled HTN, a data-driven approach integrating traditional (logistic regression) and machine learning methods was employed. Variable selection was conducted using Least Absolute Shrinkage and Selection Operator (LASSO) regression, alongside Random Forest, and XGBoost with interpretability assessed via SHapley Additive Explanations (SHAP) values. All analyses were conducted using R 4.4.1 (R Core Team, Vienna, Austria).

### LASSO regression analysis

We used a LASSO regression for identifying and excluding factors unrelated to the evaluated outcome. The alpha was set at 1 and the binomial family was employed. This approach allowed us to reduce overfitting and to simplify the model.

To determine the optimal penalty parameter, we applied 10-fold cross-validation. Variables with zero coefficients at this λ value were excluded from the model.

### Random Forest algorithm

We employed the Random Forest algorithm to predict the binary outcome. The predictor variables were those identified in LASSO regression.

The model was trained on the training dataset using the specified parameters: number of trees (ntree), 500; number of variables to consider at each split (mtry), 3; node size (nodesize), 1 for classification tasks. Variable importance was assessed based on the percentage increase in mean squared error (%IncMSE) and the increase in node purity (IncNP). These parameters were selected to balance model performance and interpretability. To assess the model’s performance, the dataset was split into training (80%) and testing (20%) subsets.

### XGBoost implementation.

The model was trained using XGBoost, a highly efficient and scalable ensemble learning algorithm designed to optimize predictive accuracy. The predictor variables were also those identified in LASSO regression.

The model was optimized for binary classification using a logistic objective function. A maximum tree depth of six was set to balance model complexity and generalizability, preventing overfitting while allowing sufficient feature interactions. A learning rate (eta) of 0.3 was applied to regulate the contribution of individual trees within the boosting process, ensuring stable learning across iterations. The model underwent 100 boosting rounds, refining predictions through multiple tree-based adjustments.

To ensure reproducibility, a fixed random seed (123) was established, allowing for consistent results across multiple training runs. Model development followed a data partitioning strategy, whereby 80% of the dataset was allocated for training, while the remaining 20% was reserved for independent evaluation, ensuring robust performance assessment.

### SHAP analysis

To enhance the interpretability of the XGBoost model, SHAP (was employed, providing a measure of the marginal contribution of each predictor toward the likelihood of uncontrolled HTN. This approach enables a more transparent understanding of feature importance, allowing for meaningful clinical interpretations.

### Logistic regression

To assess the specific effect of the evaluated exposures on the odds of having uncontrolled HTN, a multiple logistic regression model was built to compute odds ratios (OR) and 95% confidence intervals (CI). We included all variables identified as relevant in the LASSO regression.

### Performance measures

The performance of the models was assessed in terms of area under curve (AUC), sensitivity, specificity, precision (positive predictive value), accuracy and F1 score (F-measure).

### Ethical considerations

Participants in ENSANUT 2022 provided informed written consent before their participation. Since this study utilized de-identified data, no additional consent was necessary. The dataset is publicly available, and ethical approval for secondary analyses was not required.

## Results

Data from 1,308 participants were analyzed, with the majority (69.8%) being female. The mean age was 61.1 years (± 13.0) and ranged (interquartile range) from 52 to 70 years. Uncontrolled HTN was observed in 30.3% (n = 396/1,308) of the participants.

Table 1 summarizes the characteristics of the participants analyzed based on their blood pressure control status. Those with uncontrolled HTN were generally older, had a lower socioeconomic status, and had been diagnosed with the condition for a longer period compared to those with controlled HTN.

**Table 1:** Characteristics of the study sample for selected variables by blood pressure status, Mexico 2022.

	Uncontrolled blood pressure		p
	No (n = 912)	Yes (n = 396)	
Sex			
Female	650 (71.3)	263 (66.4)	0.079
Male	262 (28.7)	133 (33.6)	
Age (years, mean ± SD)	60.1 ± 13.1	63.4 ± 12.3	< 0.001
Age group (years)			
20–49	204 (22.4)	55 (13.9)	0.001
60–64	337 (36.9)	146 (36.9)	
65 or more	371 (40.7)	195 (49.2)	
Socioeconomic status			
Low	248 (27.2)	141 (35.6)	0.003
Middle	320 (35.1)	138 (34.9)	
High	344 (37.7)	117 (29.5)	
Diabetes mellitus history			
No	604 (66.2)	248 (62.6)	0.209
Yes	308 (33.8)	148 (37.4)	
Source of medical care			
Social security institutions	476 (52.2)	190 (48.0)	0.270
Public health sector	170 (18.6)	87 (22.0)	
Private care	250 (27.4)	108 (27.2)	
Other	16 (1.8)	11 (2.8)	
Years since HTN diagnosis			
≤ 5	419 (46.0)	142 (35.86)	0.002
6–10	211 (23.1)	101 (25.51)	
> 10	282 (30.9)	153 (38.64)	
Adherence to a nutritional plan			
No	631 (69.2)	285 (72.0)	0.313
Yes	281 (30.8)	11 (28.0)	
Engagement in physical activity			
No	757 (83.0)	331 (83.6)	0.796
Yes	155 (17.0)	65 (16.4)	

Abbreviations: SD, standard deviation; HTN, hypertension.

Note: 1) Uncontrolled blood pressure (systolic pressure ≥ 130 mmHg or diastolic pressure ≥ 80 mmHg) was defined as the average of the second and third readings, taken 2–3 minutes apart, following an initial reading obtained after at least 5 minutes of rest; 2) Total counts and relative frequencies (%) are presented unless otherwise specified as the arithmetic mean and standard deviation; 3) The p-value from chi-squared or t-tests is presented as appropriate.

### LASSO regression analysis

**Fig 1:**
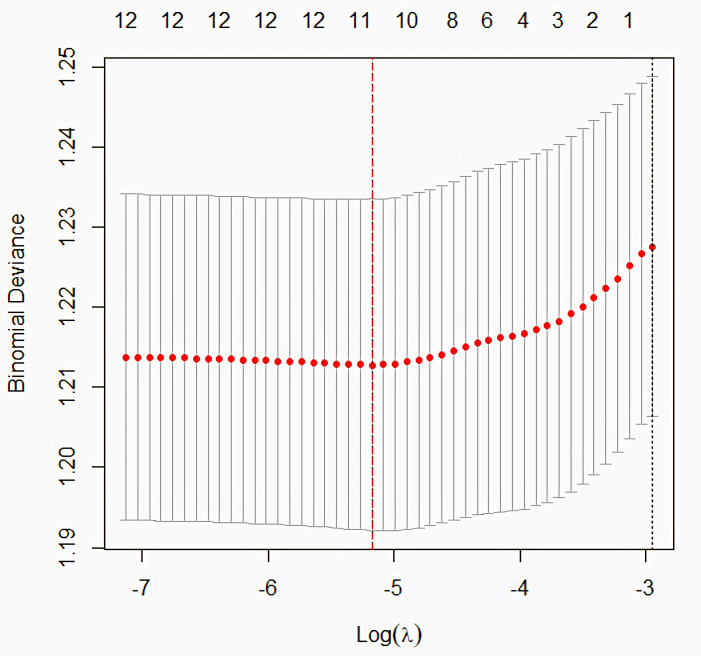
Cross-validation curve for LASSO regression model selection. Abbreviations: LASSO, Least Absolute Shrinkage and Selection Operator.

Fig 1 shows the cross-validation curve for a LASSO regression model, illustrating the relationship between the penalty term (λ) and the model’s cross-validation error. The optimal fit was selected at λ = 0.0056528.

**Fig 2:**
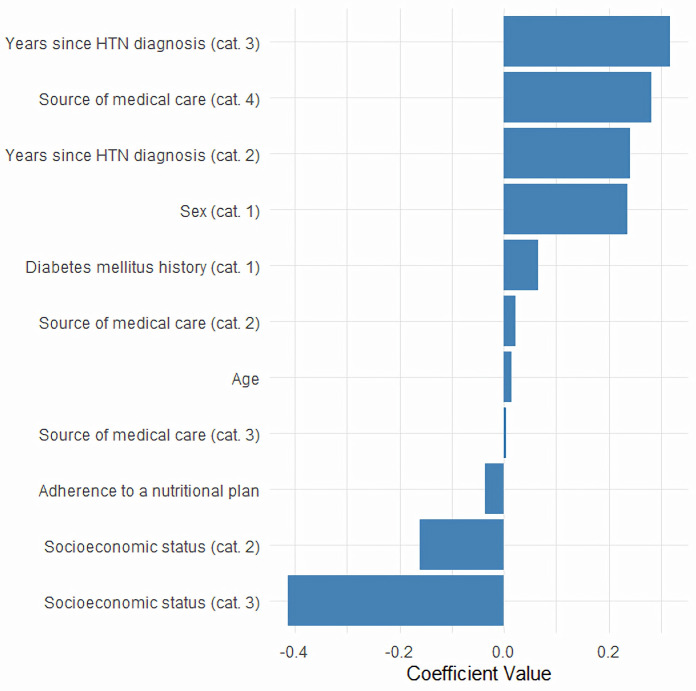
Adjusted LASSO coefficients, Mexico 2022. Abbreviations: LASSO, Least Absolute Shrinkage and Selection Operator; Cat, category; HTN, arterial hypertension. Notes: 1) The intercept LASSO coefficient was −1.75; 2) Socioeconomic status was categorized as low (1), middle (2), and high (3); 3) Source of medical care was categorized as social security institutions (1), public health sector (2), private care (3), and other (4); 4) Years since HTN diagnosis was categorized as ≤ 5 years (1), 6–10 years (2), and > 10 years (3); 5) Sex was categorized as female (0) and male (1); 6) Diabetes mellitus history was categorized as No (0) and Yes (1); 7) Engagement in physical activity was omitted because its coefficient was 0 at λ = 0.0056528.

All analyzed variables had non-zero coefficients at the selected λ, except for engagement in physical activity, which was excluded from subsequent models (Fig 2). Positive coefficients indicate an increased likelihood of poor blood pressure control, while negative coefficients suggest a protective effect or a lower likelihood of poor control.

### Random forest

**Fig 3:**
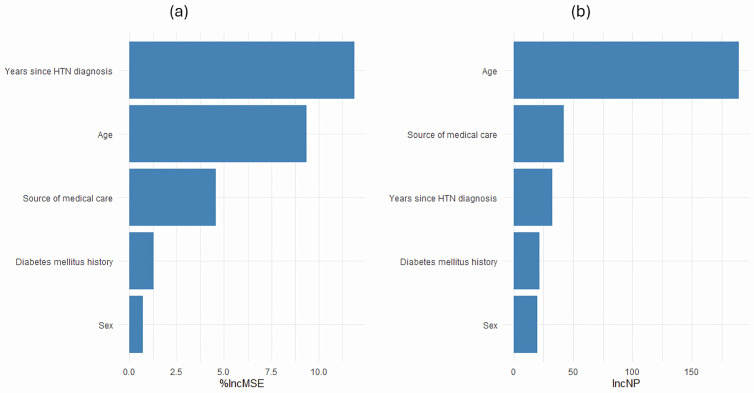
a–b. Percentage increase in mean squared error (%IMSE) (a) and the increase in node purity (IncNP) (b) by the Random Forest algorithm, Mexico 2022.

As shown in Fig 3a–b, the Random Forest algorithm identified key predictors of HTN control. Years since HTN diagnosis (11.9), age (9.4), and source of medical care (4.6) had the highest %IncMSE values, indicating their contribution to improving the model’s accuracy. For the IncNP, the most relevant variables were age (189.8), source of medical care (42.1), and years since HTN diagnosis (32.9), underscoring the importance of demographic and healthcare-related factors in this context.

### SHAP analysis in XGBoost

The SHAP values provide a measure of each feature’s impact on model decision-making. The estimated mean SHAP values indicated that age was the most influential predictor (0.115), reinforcing established epidemiological evidence that older individuals face a higher likelihood of blood pressure dysregulation.

**Fig 4:**
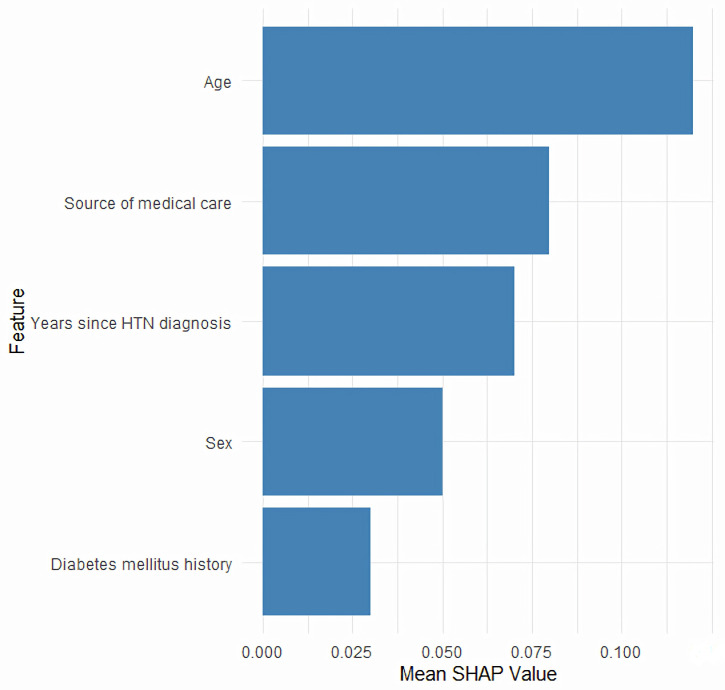
SHAP analysis in XGBoost, Mexico 2022. Abbreviations: SHAP, SHapley Additive exPlanations; XGBoost, Extreme Gradient Boosting.

The source of medical care (mean SHAP = 0.065) and years since HTN diagnosis (mean SHAP = 0.055) followed as key contributors. The influence of sex (0.035) aligns with prior findings on gender disparities in HTN prevalence (Fig 4).

### Logistic regression

In the multiple unconditional logistic regression model (Table 2), males were more likely (OR = 1.37, 95% CI 1.06–1.79) to have uncontrolled HTN compared to females. Increasing age was also associated with higher odds of uncontrolled disease, with a 1% increase per additional year (OR = 1.01, 95% CI 1.01–1.02). High socioeconomic status showed a protective effect compared to low socioeconomic status (OR = 0.58, 95% CI 0.43–0.80), which was stronger than the middle level, though the latter was not statistically significant (OR = 0.76, 95% CI 0.56–1.02). Participants with a longer duration of HTN were more likely to have uncontrolled HTN compared to those diagnosed within the past 5 years (6–10 years OR = 1.46, 95% CI 1.07–2.01; > 10 years OR = 1.56, 95% CI 1.16–2.10). No other significant associations were observed.

**Table 2:** Factors associated with uncontrolled HTN, Mexico 2022.

	OR (95% CI), p	
	Bivariate analysis	Multiple analysis
Sex		
Female	1.00	1.00
Male	1.25 (0.97–1.62), 0.079	1.37 (1.05–1.78), 0.019
Age (years)	1.02 (1.01–1.03), < 0.001	1.01 (1.01–1.02), 0.004
Socioeconomic status		
Low	1.00	1.00
Middle	0.76 (0.60–1.01), 0.059	0.76 (0.56–1.02), 0.070
High	0.60 (0.45–0.80), 0.001	0.58 (0.42–0.80), 0.001
Diabetes mellitus history		
No	1.00	1.00
Yes	1.17 (0.92–1.50), 0.209	1.13 (0.88–1.46), 0.331
Source of medical care		
Social security institutions	1.00	1.00
Public health sector	1.28 (0.94–1.75), 0.114	1.09 (0.79–1.51), 0.606
Private care	1.08 (0.82–1.43), 0.582	1.12 (0.84–1.50), 0.442
Other	1.72 (0.78–3.78), 0.175	1.63 (0.73–3.63), 0.232
Years since HTN diagnosis		
≤ 5	1.00	1.00
6–10	1.41 (1.04–1.91), 0.026	1.46 (1.07–2.01), 0.018
> 10	1.60 (1.22–2.11), 0.001	1.56 (1.16–2.10), 0.004
Adherence to a nutritional plan		
No	1.00	1.00
Yes	0.87 (0.67–1.13), 0.313	0.89 (0.67–1.18), 0.424

Abbreviations: HTN, hypertension; OR, odds ratio; CI, confidence interval. Note: 1) Unconditional logistic regression models were used to obtain OR and 95% CI; 2) Estimates from multiple analysis were adjusted by all variables listed in the table.

### Performance comparison

Among the predictive models evaluated (Table 3), Random Forest had the highest AUC (0.75, 95% CI 0.72–0.77), reinforcing its superior ability to distinguish between controlled and uncontrolled HTN. In contrast, Logistic Regression showed moderate discrimination (AUC = 0.61, 95% CI 0.59–0.64), while XGBoost documented the lowest predictive performance (AUC = 0.54, 95% CI 0.49–0.60). These findings underscore the advantages of ensemble learning techniques, particularly Random Forest, in optimizing classification performance.

**Table 3:** Performance metrics of the employed modeling methods, Mexico 2022.

	Point estimate (95% CI)					
	AUC	Precision	Sensitivity	Specificity	Accuracy	F1 Score
Random Forest	0.75 (0.72–0.77)	0.94 (0.91–0.97)	0.51 (0.46–0.55)	0.98 (0.97–0.99)	0.84 (0.82–0.86)	0.659
XGBoost	0.54 (0.49-0.60)	0.42 (0.28-0.57)	0.23 (0.14-0.33)	0.85 (0.80-0.90)	0.65 (0.60-0.72)	0.294
Logistic Regression	0.61 (0.59–0.64)	0.70 (0.68–0.72)	0.98 (0.98–0.99)	0.03 (0.02–0.03)	0.70 (0.67–0.72)	0.818

Abbreviations: AUC, area under curve, CI, confidence interval.

Precision and sensitivity are critical measures of a model’s reliability in correctly identifying cases of uncontrolled HTN. Random Forest achieved the highest precision (0.94, 95% CI 0.91–0.97), minimizing false positives, whereas Logistic Regression demonstrated high sensitivity (0.98, 95% CI 0.98–0.99), effectively identifying cases but with markedly low specificity (0.03, 95% CI 0.02–0.03). This tradeoff limits Logistic Regression’s applicability in distinguishing between controlled and uncontrolled cases with confidence.

In terms of overall model performance, Random Forest exhibited the highest accuracy (0.84, 95% CI 0.82–0.86), reinforcing its predictive reliability across various scenarios. XGBoost showed lower accuracy (0.65, 95% CI 0.60–0.72), primarily due to its reduced sensitivity, whereas Logistic Regression achieved moderate accuracy (0.70, 95% CI 0.67–0.72), despite its low specificity. Additionally, Random Forest maintained a competitive F1 score of 0.659, further supporting its robustness and suitability for identifying predictors of uncontrolled HTN in clinical and epidemiological research.

## Discussion

Three in ten participants had uncontrolled HTN despite receiving medical care, underscoring a major public health concern. Older age and longer duration since diagnosis were associated with poor blood pressure control, as identified by both logistic regression and data-driven algorithms.

Published research shows that older adults with HTN are more likely to have uncontrolled blood pressure, even with ongoing medical treatment [18]. This is linked to factors such as increased arterial stiffness with age [19], coexisting health conditions [20], and reduced effectiveness of antihypertensive medications due to changes in drug metabolism [21].

In our Random Forest algorithm, age had the highest INP, indicating it was the most significant predictor of uncontrolled blood pressure. This means that participants’ age enhanced the model’s predictive performance, highlighting its importance as a key feature. Similarly, in a traditional epidemiological approach using multiple logistic regression, each additional year of age increased the odds of uncontrolled HTN by approximately 1% (OR = 1.01, 95% CI 1.01–1.02). Age-related changes in the risk of high blood pressure have been previously reported [22].

The duration of HTN since its diagnosis was also significant in the study sample. This finding is plausible and may be linked to structural changes in blood vessels and the heart, which can complicate management [23]. The logistic regression model showed a positive gradient in the uncontrolled HTN odds and, when compared to those with a shorter disease evolution (< 5 years), those with 6–10 years or > 10 years

Among the predictive models evaluated, Random Forest demonstrated superior performance, balancing high predictive accuracy, sensitivity, and precision while maintaining a reasonable specificity. The model achieved an AUC of 0.75 (95% CI 0.72–0.77), indicating its robustness in distinguishing between controlled and uncontrolled HTN. Additionally, its precision (0.94, 95% CI 0.91–0.97) was notably high, ensuring reliable identification of at-risk individuals, while its sensitivity (0.98, 95% CI 0.97–0.99) confirmed its effectiveness in detecting cases of uncontrolled HTN.

A longer duration of HTN has consistently been associated with an increased risk of uncontrolled blood pressure, leading to heightened cardiovascular morbidity and mortality. Published research indicates that individuals with HTN for more than 10 years have higher odds of poor blood pressure control compared to those with a shorter disease duration [24]. This association is attributed to progressive vascular remodeling, arterial stiffness, and diminished responsiveness to antihypertensive therapy over time [25]. Additionally, prolonged HTN duration independently contributes to adverse cardiovascular outcomes, even after adjusting for blood pressure levels [25].

The presented results highlight the need for tailored interventions targeting older adults, and those with longer durations since diagnosis. Efforts to improve blood pressure control should prioritize improving healthcare access and addressing socioeconomic disparities, as these were strongly associated with uncontrolled HTN. Interventions promoting adherence to nutritional plans and addressing comorbidities like diabetes could also play a critical role in improving outcomes.

HTN remains a major global health challenge, affecting over 1.3 billion individuals worldwide [26]. Despite the availability of pharmacological treatments, a substantial proportion of patients fail to achieve adequate blood pressure control, increasing the risk of stroke, cardiovascular disease, and chronic kidney dysfunction. In Mexico, approximately 30 million adults live with HTN, with nearly half unaware of their condition, leading to frequent hospitalizations and severe complications [27].

The economic burden of HTN is high, particularly in low- and middle-income countries, where healthcare systems face significant financial strain. Studies indicate that the management of HTN and its complications accounts for a substantial portion of national healthcare expenditures, exacerbating resource limitations [28]. Additionally, social determinants of health, including access to healthcare, medication adherence, and lifestyle factors, play a crucial role in disease progression and treatment outcomes [29].

The predictive strength of data-driven models suggests that may complement traditional epidemiological methods by offering enhanced accuracy in identifying individuals at risk. However, the complexity of these algorithms may pose challenges for implementation in routine clinical practice. Efforts to integrate machine learning approaches into healthcare systems must prioritize usability and accessibility to maximize their impact.

Several limitations should be acknowledged. First, the study’s cross-sectional design prevents the establishment of causal relationships between the identified predictors and uncontrolled HTN. Second, self-reported data on HTN diagnosis and medication use may introduce recall bias, potentially affecting the accuracy of reported treatment patterns.

Third, medication adherence and sustained healthcare access were not explicitly measured. Although self-reported medication use was included, detailed adherence levels remain unknown. To address this gap, future studies should incorporate direct adherence metrics, improving HTN risk assessments. The usual source of medical care was considered as a predictor, providing indirect insight into accessibility.

Fourth, secondary causes of HTN, including renal and endocrine disorders, were not assessed due to data limitations in ENSANUT 2022. Fifth, body mass index and obesity status were excluded from the analysis because their inclusion would have reduced the sample size by nearly 20%. Given the study’s focus on an exploratory data-driven model, maintaining a larger dataset was essential to preserve model stability and predictive accuracy. While obesity is a well-established determinant of blood pressure control, future studies should examine its role within data-driven predictive frameworks, balancing dataset constraints with methodological rigor.

Finally, data were collected from a single population, which may limit the generalizability of the findings to broader demographic or geographic groups. Future research should investigate diverse populations to validate and expand the applicability of these results.

## Conclusion

This study highlights the effectiveness of combining traditional statistical methods with AI-assisted data-driven algorithms to identify key predictors of uncontrolled hypertension. By addressing the modifiable and potentially modifiable risk factors identified through LASSO regression, Random Forest, and XGBoost with SHAP, public health strategies can be more precisely tailored to at-risk populations, improving targeted interventions. Future research should prioritize longitudinal designs and data-driven interventions, optimizing predictive analytics to refine precision prevention and personalized treatment efforts. These approaches will enhance the ability to identify high-risk individuals earlier, fostering more effective public health policies and clinical decision-making.
